# Shotgun metagenomics of soil invertebrate communities reflects taxonomy, biomass, and reference genome properties

**DOI:** 10.1002/ece3.8991

**Published:** 2022-06-06

**Authors:** Alexandra Schmidt, Clément Schneider, Peter Decker, Karin Hohberg, Jörg Römbke, Ricarda Lehmitz, Miklós Bálint

**Affiliations:** ^1^ Senckenberg Biodiversity Climate Research Center Frankfurt am Main Germany; ^2^ Biology Department J.W. Goethe University Frankfurt am Main Germany; ^3^ Loewe Center for Translational Biodiversity Genomics (LOEWE‐TBG) Frankfurt am Main Germany; ^4^ Limnological Institute (Environmental Genomics) University of Konstanz Konstanz Germany; ^5^ Soil Zoology Department Senckenberg Museum of Natural History Görlitz Görlitz Germany; ^6^ Blumenstr. 5 Görlitz Germany; ^7^ ECT Oekotoxikologie GmbH Flörsheim am Main Germany; ^8^ Institute for Insect Biotechnology Justus Liebig University Gießen Germany

**Keywords:** biomonitoring, eukaryotes, genome completeness, genome size, invertebrates, shotgun metagenomics, species composition, taxonomic bias

## Abstract

- Metagenomics – shotgun sequencing of all DNA fragments from a community DNA extract – is routinely used to describe the composition, structure, and function of microorganism communities. Advances in DNA sequencing and the availability of genome databases increasingly allow the use of shotgun metagenomics on eukaryotic communities. Metagenomics offers major advances in the recovery of biomass relationships in a sample, in comparison to taxonomic marker gene‐based approaches (metabarcoding). However, little is known about the factors which influence metagenomics data from eukaryotic communities, such as differences among organism groups, the properties of reference genomes, and genome assemblies.
- We evaluated how shotgun metagenomics records composition and biomass in artificial soil invertebrate communities at different sequencing efforts. We generated mock communities of controlled biomass ratios from 28 species from all major soil mesofauna groups: mites, springtails, nematodes, tardigrades, and potworms. We shotgun sequenced these communities and taxonomically assigned them with a database of over 270 soil invertebrate genomes.
- We recovered over 95% of the species, and observed relatively high false‐positive detection rates. We found strong differences in reads assigned to different taxa, with some groups (e.g., springtails) consistently attracting more hits than others (e.g., enchytraeids). Original biomass could be predicted from read counts after considering these taxon‐specific differences. Species with larger genomes, and with more complete assemblies, consistently attracted more reads than species with smaller genomes. The GC content of the genome assemblies had no effect on the biomass–read relationships. Results were similar among different sequencing efforts.
- The results show considerable differences in taxon recovery and taxon specificity of biomass recovery from metagenomic sequence data. The properties of reference genomes and genome assemblies also influence biomass recovery, and they should be considered in metagenomic studies of eukaryotes. We show that low‐ and high‐sequencing efforts yield similar results, suggesting high cost‐efficiency of metagenomics for eukaryotic communities. We provide a brief roadmap for investigating factors which influence metagenomics‐based eukaryotic community reconstructions. Understanding these factors is timely as accessibility of DNA sequencing and momentum for reference genomes projects show a future where the taxonomic assignment of DNA from any community sample becomes a reality.

## Graphical abstract

Shotgun metagenomics is increasingly applicable to study eukaryotic community composition. Theoretically, metagenomics should inform about species’ biomass, since it omits a taxonomically biased PCR step. Here, we evaluate how well metagenomics records composition and biomass in mock communities, and how its performance is affected by reference genome properties.

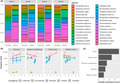

## 1 INTRODUCTION

Biodiversity research, and particularly the investigation of hard‐to‐observe ecological communities, increasingly relies on DNA‐ and RNA‐based tools such as metabarcoding (Taberlet et al., 2018), metagenomics (Arribas et al., 2020), or metatranscriptomics (Cristescu, 2019). There are several preconditions to the use of these tools for generating datasets on ecological community composition: nucleotide sequence databases must exist (Hebert et al., 2003; Margaryan et al., 2021) with curated taxonomic links (Schenk et al., 2017) for taxonomic identification of DNA or RNA sequences. Laboratory experimental designs must also be robust, with excellent guidance already existing (Zinger et al., 2019). However, if preconditions are met, molecular tools can provide data on the composition and structure of ecological communities, even if they are made up of very small, diverse, and difficult to identify species.

Molecular tools to monitor communities can be time and cost efficient when compared to conventional, observation‐based studies, where species are morphologically identified and counted to document abundances (Serrana et al., 2019). This is especially the case when observed communities are species rich, and when many community samples need to be processed simultaneously (Bálint et al., 2018). This needs expertise on certain taxonomic groups, which makes it difficult for one researcher to acquire composition data. Molecular tools overcome this issue as whole communities, containing various taxonomic groups, can be identified at once, in many samples run in parallel (e.g., Zinger et al., 2019). There are two main approaches to the molecular biomonitoring of communities: metabarcoding and metagenomics. Metabarcoding uses high‐throughput sequences of taxonomic marker genes (“barcodes”) which are PCR amplified from a community DNA extract. Metabarcoding is becoming a standard tool in biodiversity research (Bálint et al., 2018; Bohmann et al., 2021; Compson et al., 2020; Creer et al., 2016; Jarman et al., 2018; Lindahl et al., 2013; Taberlet et al., 2012). Its use is supported by several years of research in distinct organism groups (Taberlet et al., 2018), and the availability of barcode databases (Hebert et al., 2003; Nilsson et al., 2019). However, metabarcoding has an important long‐known drawback: it relies on the amplification of a marker gene (Taberlet et al., 2012). This can result in biases in species recovery from the resulting sequence data: several species might be completely missed as false negatives if metabarcoding PCR primers poorly match binding sites in their genomes in a phenomenon known as PCR bias (Zinger et al., 2019). Sometimes PCR bias is not sufficiently strong to completely miss species, but primer mismatch still causes a less efficient amplification compared to other species, resulting in distortions of the original biomass–sequencing read relationships for certain taxa (Piñol et al., 2019). However, the amplification step solves two important issues: one can effectively target the taxonomic groups of interest (e.g., insects) and avoid others (e.g., microorganisms), and small or rare organisms with low amounts of DNA can still be recorded. Metagenomics randomly sequences all DNA fragments from a community DNA extract, generally without enrichment of certain parts of the genome. It is more quantitative than metabarcoding, since it skips the potentially biased PCR amplification step of taxonomic marker genes (Bista et al., 2018), and consequently, may provide more detailed insights into the biomass ratios of different species (Peel et al., 2019). Biomass ratios are important for ecological studies as the importance of species in a community is often directly related to its abundance or biomass (Naeem et al., 2009). Biomonitoring schemes frequently rely on indices of environmental quality which are computed from species identities, and abundance or biomass ratios (Bennion & Battarbee, 2000/60/EC of the European Parliament and of the Council of 23 October 2000). However, metabarcoding can provide limited information on this given biases caused by the PCR step (Aird et al., 2011), and currently this limits its use in applied biomonitoring (Hering et al., 2018). In metagenomics, a random selection of DNA fragments is sequenced from the DNA extracts, resulting in a less biased representation of the community in the sequence data. The omission of the PCR step makes metagenomics lab work conceptually and technically simpler. From metagenomic studies of microbial mock communities we know that several factors, such as taxonomic identity (Schiebelhut et al., 2017) or the genome properties of involved species (Beszteri et al., 2010), have an impact on biomass representation through metagenomic reads. However, these effects are so far not investigated in metagenomics studies of eukaryotes, at least to our best knowledge. The taxonomic assignment of metagenomic sequences needs genome databases, and consequently, metagenomics is more frequently applied on microbial communities, where more complete genomic resources are available (Parks et al., 2020). There are several approaches to circumvent this limitation, from mitogenomes (Arribas et al., 2020) to shallow genome sequencing (Bohmann et al., 2020). As genome sequencing technologies mature, the generation of reference genomes for all eukaryotes receives increasing attention (Lewin et al., 2018). However, the technical issues affecting metagenomics, such as species identification success, read–biomass relationships, the effects of different DNA extraction techniques, and the effects of reference genome properties used for taxonomic identification of metagenomic reads are much less investigated than issues affecting metabarcoding, at least for eukaryotes. Approaches to metagenomic read classification need also be evaluated for eukaryotes, since there are several algorithms available, and these algorithms can be adjusted to allow for more or less base dissimilarities among query and database sequences (Altschul et al., 1990; Wood et al., 2019).

Soil invertebrate communities are diverse, with high numbers and often high biomass of taxa (FAO, 2020). Most soil invertebrate species are very small, with body lengths below 1 mm (Orgiazzi et al., 2016). Despite their small size, invertebrates are important for soil health (Kibblewhite et al., 2008), and high biomass species strongly contribute to soil functioning (van den Hoogen et al., 2019). However, ecological work and biomonitoring of these communities are difficult: taxonomic identification needs very specialized expertise (Lehmitz & Decker, 2017). This makes morphology‐based identification efforts unfeasible for the large sample numbers needed by most community ecology and biomonitoring efforts. The increasing availability of reference genomes (Lewin et al., 2022) makes metagenomics a promising approach to describe and monitor community composition and species biomass ratios of soil invertebrates. Here, we evaluate the performance of metagenomics in species identification in artificially composed (mock) communities of soil invertebrates. We also evaluate how well metagenomics reflects biomass ratios of species. We use a large collection of soil invertebrate genomes to taxonomically assign metagenomic reads. We investigate the effects of metagenomic classification thresholds on correct and false identification. We evaluate the relationship between biomass and reads, and how this relationship is influenced by taxonomy and by the properties of the genome assemblies used for taxonomic assignments. Finally, we evaluate how different sequencing efforts influence metagenomics results, as this strongly influences the economics of eukaryotic metagenome sequencing.

## 2 MATERIAL AND METHODS

### 2.1 Mock community construction

**FIGURE 1:**
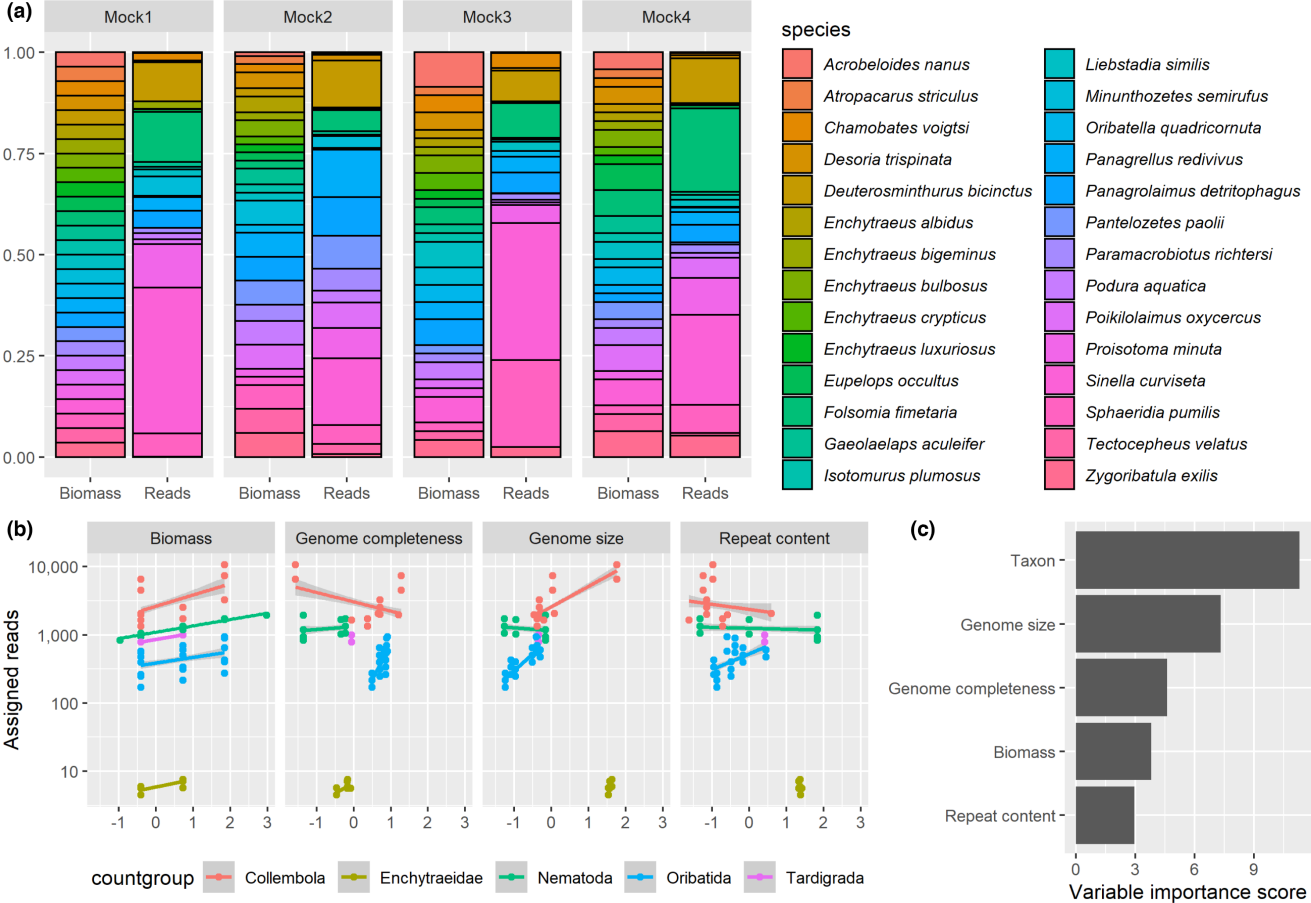
(a) Ratios of species biomass and sequencing reads assigned to these species in the four mock communities types. (b) GLM‐predicted effects of biomass, genome completeness, genome size, and repeat content on taxonomically assigned metagenomic reads. (c) Relative importance of GLM predictor variables

We constructed mock communities from 28 soil invertebrate species from six major taxonomic groups at the Senckenberg Museum of Natural History Görlitz. Specimens were either freshly collected and stored in 96% undenatured ethanol (Collembola, Gamasida, and Oribatida), or they came from breeding cultures (Enchytraeidae, Nematoda, and Tardigrada). Mock communities were composed from individual animals and animal fragments (and not from DNA extracts). These individuals/fragments were placed into single test tubes per mock community to create bulk community samples. Four different mock types were designed (Figure 1a, Table 1). We varied the total body volume (the sum of body volumes of all individuals of a species) across the four mock communities, meaning the total volume as well as species volumes differ in all four set‐ups. The mocks contained species with very small body volumes (Nematoda, Tardigrada) and larger species (Collembola, Gamasida, and Oribatida). Enchytraeidae represent the largest taxon in this study (Table 1). We used body volume as a proxy of biomass, and refer to it as biomass throughout the text. In the first mock, all species were represented with equal biomass. In the second mock, each of the small species had two to five times more biomass compared to any large species. In the third mock, a part of small species (7 of 11) had larger biomass (two to four times than any large species). In the fourth mock, most small species had more biomass than large species, but some large species also had high biomass. All four mock types were replicated three times: we attempted to reproduce the same biomass ratios among the species. This altogether gave us 12 mock communities.

**TABLE 1:** Composition of mock communities. For species where different developmental stages were available, individuals of different sizes were used to achieve the necessary biomass [adults + juveniles, e.g., *Paramacrobiotus richtersi* in mock 1: 4 + 1]. Mock 1: all species have equal biomass; mock 2: small species have higher biomass; mock 3: some, but not all small species have higher biomass than large species; mock 4: some small and some large species both have higher biomass than other small and large species

Taxon	Mean body length (µm)	Body volume (10^−6^ µm^3^)	Number of individuals			
			Mock 1	Mock 2	Mock 3	Mock 4
Tardigrada						
*Paramacrobiotus richtersi* (Murray, 1911)	700	12.1	4+1	9	0+9	2+5
Nematoda						
*Acrobeloides nanus* (de Man, 1880)	340	0.15	355	1775	1420	710
*Panagrolaimus detritophagus* (Fuchs, 1930)	380	0.10	521	1562	1562	521
*Panagrellus redivivus* (Linnaeus, 1767)	620	0.28	190	570	380	190
*Poikilolaimus oxycerca* (de Man, 1895)	930	0.98	54	162	54	162
Collembola						
*Sphaeridia pumilis* (Krausbauer, 1898)	300	5.7	9	9	37	9
*Proisotoma minuta* (Tullberg, 1871)	880	11.0	5	4	5	5
*Podura aquatica* (Linnaeus, 1758)	560	13.9	4	12	8	8
*Desoria trispinata* (MacGillivray, 1896)	1090	17.3	3	6	6	6
*Isotomurus plumosus* (Bagnall, 1940)	1250	31.0	2	2	2	2
*Deuterosminthurus bicinctus* (Koch, 1840)	730	36.1	1+1	1+1	1+1	1+1
*Sinella curviseta* (Brook, 1882)	1090	44.1	1	1	4	4
*Folsomia fimetaria* (Linnaeus, 1758)	1400	53.2	1	1	2	3
Oribatida						
*Tectocepheus velatus* (Michael, 1880)	240	4.8	11	33	11	22
*Minunthozetes semirufus* (C. L. Koch, 1841)	280	5.6	9	28	19	10
*Pantelozetes paolii* (Oudemans, 1913)	340	12.9	4	12	4	8
*Zygoribatula exilis* (Nicolet, 1855)	360	13.7	4	12	8	12
*Chamobates voigtsi* (Oudemans, 1902)	300	15.9	3	3	7	3
*Atropacarus striculus* (C. L. Koch, 1835)	440	27.1	2	2	2	2
*Liebstadia similis* (Michael, 1888)	470	35.5	2	1	5	3
*Eupelops occultus* (C. L. Koch, 1835)	410	46.5	1	1	1	3
*Oribatella quadricornuta* (Michael, 1880)	560	50.8	1	1	2	2
Gamasida						
*Gaeolaelaps aculeifer* (Canestrini, 1883)	700	22.0	2+1	5	2+1	5+6
Enchytraeidae						
*Enchytraeus bulbosus* (Nielsen & Christensen, 1963)	4000		Fragments			
*Enchytraeus albidus* (Henle, 1837)	2500					
*Enchytraeus luxuriosus* (Schmelz & Collado, 1999)	10500					
*Enchytraeus bigeminus* (Nielsen & Christensen, 1963)	6500					
*Enchytraeus crypticus* (Westheide & Graefe, 1992)	7500					

We used different formulas for body volume approximation. For Collembola, we estimated body volumes as ellipsoid volumes (*V* (µm³) = 1.33 × *π* × *a* × *b* × *c* × 10^−6^, where *a*, *b*, and *c* are axis lengths in µm). For Oribatida, Gamasida, and Enchytraeidae, we estimated body volumes as cylinder volumes (*V* (µm³) = *π* × *L* × *r* ^2^ × 10^−6^, where *L* is height and *r* is radius); for Tardigrada, *V* (µm³) = *L* × *d* ^2^ × 0.785 × 10^−6^ (Hallas & Yeates, 1972); and for Nematoda, *V* (µm³) = *L* × *d* ^2^ × 0.577 × 10^−6^ was used (Andrássy, 1956). We measured the sizes of all tardigrade, enchytraeid, springtail, and mite specimens and 20 nematode specimens used in the mock community construction to obtain average body size measures. We then combined sufficient numbers of specimens, considering variation in individual sizes to achieve the biomass ratio desired in the experimental design of the mock communities (Figure 1a).

We used the tardigrade culture *Paramacrobiotus richtersi* (Murray, 1911) strain Hohberg‐99 and the following cultures of nematodes: *Acrobeloides nanus* (de Man, 1880) strain Hohberg‐99, *Panagrolaimus detritophagus* Fuchs, 1930, strain Hohberg‐07, *Panagrellus redivivus* (Linnaeus, 1767) strain König‐18, and *Poikilolaimus oxycerca* (de Man, 1895) strain Hohberg‐01. Thousands of nematode specimens were extracted through sieves and milk filters from the culture plates into tap water. Nematode numbers and mean body volumes within the four stock solutions were then calculated by counting individuals of aliquots and measuring body length and width of 20 specimens per aliquot. After counting, we evaporated the water from each stock solution and added 96% ethanol. As enchytraeids are large compared to the other invertebrates, we used only body fragments, cutting off parts after measuring the lengths of the specimens. Tardigrades, collembolans, and mites were individually counted into the mock communities. In order to achieve the needed biomass of the respective mock type, differently sized individuals (adults and juveniles) were used. All mock community samples were stored in 2‐ml Eppendorf tubes in 96% undenatured ethanol at −20°C until sequencing.

### 2.2 Laboratory work and sequencing

We used the 12 bulk mock communities containing individuals/fragments of individuals of the 28 species for DNA extractions (four biomass ratios, each replicated three times, Table 1, Figure 1a). Before performing the DNA extraction, ethanol was evaporated in a SpeedVac Concentrator Plus (Eppendorf) to avoid losing individuals/fragments. This is especially important for potentially floating Nematoda and Tardigrada specimens. DNA was extracted with DNeasy Blood and Tissue kit (Qiagen). DNA was extracted from bulk samples. Species and specimens were mixed into mock communities prior to lysis and extraction. Replicates were extracted separately. We included a negative control into the extractions to investigate possible cross‐sample contamination. This negative control was an extraction blank without tissue. We followed the Qiagen protocol except a few modifications. We crushed specimens with pistilles in 1.5‐ml Eppendorf tubes. Before homogenizing (crushing) the tissue, we immediately added 30 µl ATL lysis buffer to inhibit the DNAse activity. Subsequently, 150 µl ATL lysis buffer and 20 µl protein kinase K were added. After vortexing and incubating (~3 h, 56°C), 20 µl RNase was added. The samples were then incubated overnight (37°C). We eluted with 50 µl AE buffer. Each resulting extract represents one replicate of the mock communities. DNA concentration was measured on NanoDrop (Thermo Fisher Scientific) and Qubit^™^ with the dsDNA BR Assay kit (Thermo Fisher Scientific). We used both tools to double check concentration measurements. Fragment length was checked on TapeStation 2200 (Agilent Technologies). Libraries were prepared with the NEB Next^®^ Ultra^™^ DNA Library Prep kit (New England Biolabs, Ipswich MA, USA) and sequenced on an Illumina NovoSeq 6000 PE150 platform at Novogene. Sequencing depth was 20 gigabase per mock community, and 1 gigabase for the negative control (2 × 150 bp, paired‐end).

### 2.3 Bioinformatics and data processing

Sequences were trimmed and quality checked with Autotrim v0.6.1 (Waldvogel et al., 2018). Autotrim relies on Trimmomatic (Bolger et al., 2014), FastQC (Andrews, 2017/2021), and MultiQC (Ewels et al., 2016). It removes Illumina sequencing adapters, performs a quality control of the reads, and combines all information into a single report. Taxonomic classification was performed with Kraken2 v2.0.8 (Wood et al., 2019) against a designated soil invertebrate genome database (GenBank Bioproject PRJNA758215). This database contains short‐read assemblies of over 270 species (FigShare doi: https://doi.org/10.6084/m9.figshare.19657647.v2, Table S1), including all species used for the mock communities. Before conducting metagenomic classification, the reference genomes were used to build a Kraken2 database with the default k‐mer size (*k* = 35). Taxonomic identification of reads was performed on 21 classification thresholds (between 0.0 and 1.0, at 0.05 increments). At each classification threshold, we accounted for possible contamination by extracting the hits of each taxon found in the negative control from the hits of that taxon in every mock community. We plotted correctly identified taxa, false negatives, and false positives against the Kraken2 classification threshold, and selected the best performing assignments for further analysis. The extraction negative control was additionally identified with the most current NCBI nt database via Kraken2 (download: March 24^th^, 2022) to analyze if other taxa than invertebrates are present. The result was visualized with KronaTools v2.7.1 (Marbl/Krona, 2015/2022).

### 2.4 Data analysis

Data analysis was conducted with R v3.6.1 in RStudio (v1.2.1335), with data formatted with tidyverse (Wickham et al., 2019). Graphs and plots were generated by using the package ggplot2 (Wickham, 2016). Unclassified reads, and classified reads representing <0.01% of the sample were removed from data. We evaluated false negatives and false positives at all 21 Kraken2 classification thresholds (FigShare doi: https://doi.org/10.6084/m9.figshare.19657647.v2).

We predicted read abundances with the total number of sequences obtained for each mock library with a generalized linear model. Initial independent variables were sequencing success, taxon group (Collembola, Enchytraeidae, Nematoda, Oribatida, Gamasida, and Tardigrada), mock species biomasses, genome completeness (measured recovered complete Benchmarking Universal Single‐Copy Orthologs, complete BUSCOs (Simão et al., 2015)), GC content, genome sizes, and repeat content. We estimated genome sizes with ModEst, a new method, which performs very well in comparison with flow cytometry measurements (Pfenninger et al., 2021). We estimated the repeat content of genomes with species‐specific repeat libraries which were constructed using an automated RepeatModeler 2.0.1 pipeline with LTR Structural discovery pipeline activated (Flynn et al., 2020). For each genome, the resulting repeat libraries were merged with the RepBase 26.05 Arthropoda‐specific section (Bao et al., 2015) and subsequently used for the annotation of repetitive elements with RepeatMasker 4.1.2‐P1 (Smit et al., 2015). First, we performed a combinatorial model selection with MuMIn (Burnham & Anderson, 2003). The best performing model based on quasi‐AIC scores can be written up as hits ~ biomass + taxon_group + missing_buscos + genome_size + repeat_content. The final model was fitted with quasi‐Poisson distribution to account for overdispersion. All predictors were scaled. Genome sizes were log‐normalized before scaling. We evaluated the relative importance of the predictors by calculating model‐specific variable importance scores in the R package vip (Greenwell & Boehmke, 2020).

We evaluated the correspondence between community composition captured by metagenomic reads and original biomass composition with redundancy analyses in vegan (Oksanen et al., 2019). We tested metagenomic hit model statistical significance with an ANOVA‐like permutation test for redundancy analysis (Legendre & Legendre, 2012).

We re‐run read taxonomic identification after subsampling raw sequences to 100,000, 500,000, 1,000,000, 5,000,000, and 10,000,000 reads with seqtk (Li, 2012/2022). We compared results of data analysis done with the complete number of reads with results obtained after analyzing 100,000 reads. This allows to evaluate how strongly sequencing effort influences metagenomic results.

## 3 RESULTS

**FIGURE 2:**
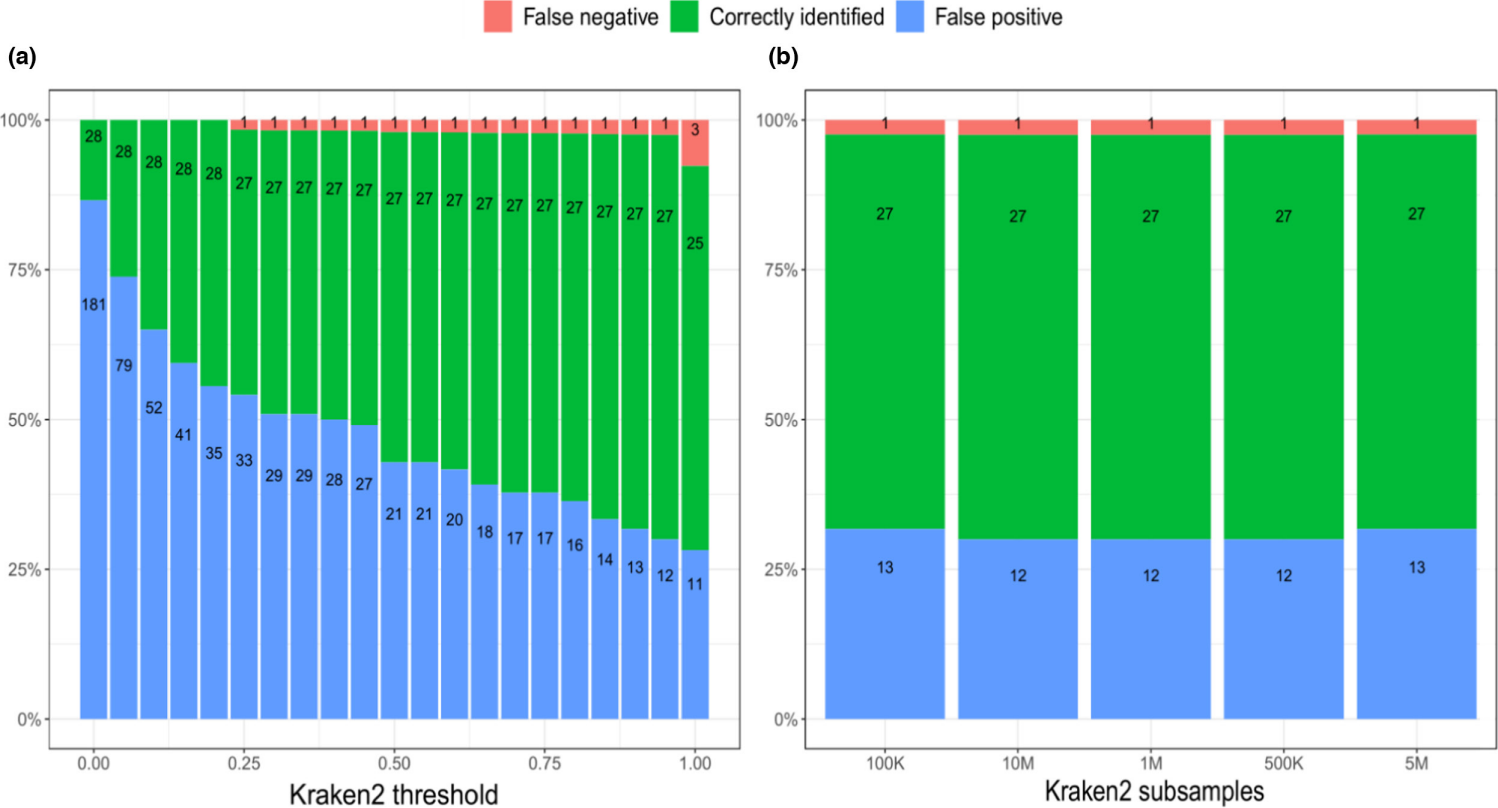
Numbers over bars represent the actual numbers of correctly identified species, and false‐negative and false‐positive identifications. (a) Species identification success along different Kraken2 classification thresholds. (b) Species identification success along different subsample sizes

The sequencing resulted in ~69 million paired‐end reads on average per mock community replicate, with a standard deviation of ~1.5 million reads (Figure 1a). Raw sequencing results are available on the European Nucleotide Archive (accession number: PRJEB45431). About 10 million reads were recorded in the negative control. Only 4% of the reads from the negative control could be classified with the NCBI nt database: 1% is classified as Eukaryota, 1% as Bacteria, and the remaining 2% as viruses, Archaea, and others (Appendix S1, https://figshare.com/articles/figure/Schmidt_et_al_Suppl_File_html/19711684/1). Of the reads passing quality filtering in the mock communities, ~95 million were assigned to taxa at a 0.95 classification threshold (Table 1). The number of correctly classified species remained stable across all classification thresholds (Figure 2a). We retained results at 0.95 as a trade‐off for correct and false classifications. Of the 28 species from the mock community, 27 were correctly identified at most classification thresholds (Figure 2a). However, the number of false‐positive classifications strongly decreased at more stringent thresholds, from 181 to 11. False positives belonged to the taxa Collembola, Oribatida, and Nematode at threshold of 0.95. The number of false‐negative classifications remained low, stable, and consistent – a single species (an oribatid mite: *Atropacarus striculus*) was missed at most classification thresholds. Missing this species was due to the stringency of the bioinformatic sequence processing: The species yielded very few sequencing reads which were then discarded during data filtering. Subsampling reads had a limited influence on taxonomic identification (Figure 2b).

**FIGURE 3:**
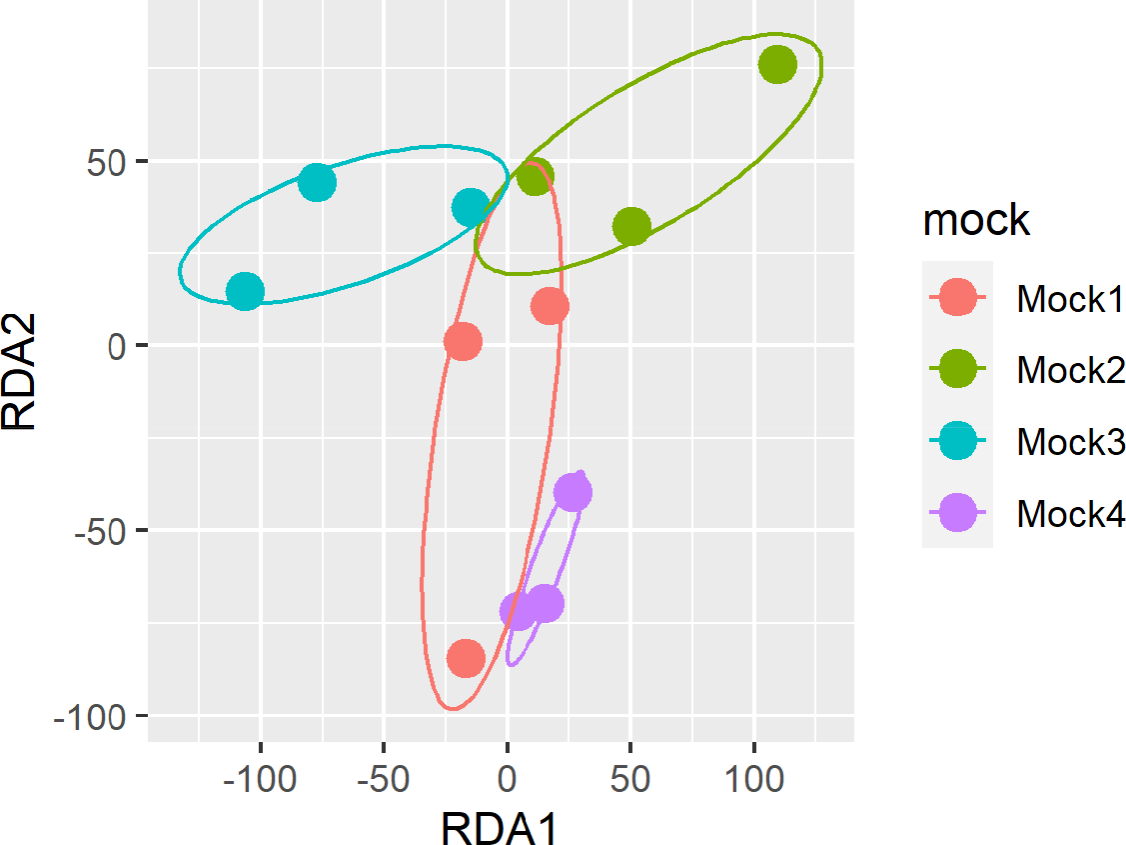
Redundancy analysis ordination of mock community replicates along the taxonomically assigned metagenomic reads

Some species consistently yielded more reads, regardless of their biomass ratios in the mocks (Figure 1a). Sequencing depth differences among mock libraries and the GC content of the genomes had little predictive effect on assigned sequencing reads, so they were discarded during model selection. The final model (Figure 1b, Table 2) showed that metagenomic sequencing success differed across the taxon groups. Compared to reads assigned to Collembola, assignment success to Tardigrada and Nematoda showed a slight but statistically insignificantly lower assignment success. Assignment success to Oribatida and Nematoda was statistically significantly lower than to Collembola (Table 2). Biomass of species was positively related to assigned metagenomic reads in all groups. Genome completeness had a statistically significant positive effect on metagenomic read assignment: overall, more reads were assigned to taxa with more complete genomes. This differed across taxon groups, as Collembola were not as much influenced by genome completeness as other taxa. Genome size had a statistically significant positive effect on metagenomic read assignment. More reads were assigned to taxa with larger genomes, regardless of the taxon group. Repeat content had a low but statistically significant effect on metagenomic read assignment (Figure 1b,c). Genome size and repeat content were collinear (Pearson *R* ^2^ = 0.56, *p* < .001). Taxon groups were the most important predictors in the model (Figure 1c). Replicates of the four mock community types were statistically significantly grouped together in the redundancy analysis (df = 3, *F* = 3.863, *p* < .001, Figure 3). Data analysis performed with 100,000 reads yielded very similar results (Figures S1 and S2).

**TABLE 2:** Model‐predicted biomass, taxon group, genome completeness, genome size, and repeat content effects on assigned metagenomic read numbers. All predictors were scaled before model fitting. Genome size was log‐normalized before scaling. Collembola served as a model intercept

	Estimate	Standard error	*t*	*p*
(Intercept)	14.047	0.132	106.498	.000
Biomass	0.192	0.054	3.582	.000
Enchytraeidae	−6.910	1.748	−3.953	.000
Nematoda	0.947	0.352	2.688	.008
Oribatida	−1.194	0.212	−5.633	.000
Tardigrada	−0.002	0.369	−0.005	.996
Genome completeness	0.599	0.122	4.891	.000
Genome size	1.238	0.160	7.761	.000
Repeat content	−0.244	0.082	−2.966	.003

## 4 DISCUSSION

We performed a shotgun metagenomic experiment on soil invertebrate mock communities of known composition. We assigned metagenomic reads to a genome database of soil invertebrates. We investigated how metagenomic reads record the presence of taxa in the mocks, whether read numbers reflect biomass. We found that almost all species from the mocks could be identified with metagenomics. We also found that metagenomic reads reflect biomass ratios among the species, but taxonomy and reference genome and assembly properties must be considered during metagenomics read assignments.

Almost all species (27/28) were consistently detected at most classification thresholds. The single false‐negative species (*A*. *striculus*) was also detected with very low read numbers, and it was missed only because of stringent quality filtering. The number of false positives was high at low classification thresholds, and rapidly dropped at higher thresholds (Figure 2a). Eleven false‐positive assignments were retained even at the highest classification threshold. Based on these results, we recommend rather stringent classification thresholds for the Kraken2‐based classification of eukaryotic metagenomic reads, although the effects of classification threshold choice should be further evaluated in different taxa.

Possible explanations include contamination and bioinformatic issues. Cross‐contamination is sometimes observed in mock metagenomes (Bista et al., 2018), but it cannot cause false positives here as all species were present in all mocks. The negative control included into the experiment also excludes cross‐contamination, as no soil invertebrate sequences were detected in the negative (Appendix S1, https://figshare.com/articles/figure/Schmidt_et_al_Suppl_File_html/19711684/1). Gut content may also result in the detection of unexpected taxa (Paula et al., 2016). However, most species used in these mocks are not predators. The predatory tardigrade *P*. *richtersi* was exclusively feeding on a nematode species which was also present in all the mock communities (*A*. *nanus*). The most likely explanation is related to aspects of the metagenomic read assignment. The first candidate is the assignment algorithm itself, although comparisons show that Kraken is conservative (Harbert, 2018). Assignment of reads to closely related taxa is an unlikely cause since 8 of the 12 false‐positive species (at 0.95 classification threshold) had no genus‐level relatives in the mocks. Assignment to genome regions highly conserved and thus similarity among species might also result in false positives. Unmasked repeats might also erroneously attract reads during the assignment. Eukaryotes are rich in low complexity regions, and cross‐assignment of these regions might be a considerable source of false positives in all eukaryotic metagenomes (Clarke et al., 2018). The effects of repeat regions in eukaryotic metagenomic assignments should be evaluated, although repeat identification is not trivial, especially for understudied taxa (Clarke et al., 2019).

The relationship between sequencing reads and the initial biomass of organisms is a central topic in the DNA‐based analysis of community composition. In theory, more shotgun metagenomic reads should be assigned to species which are represented with higher biomass in a sample. However, this relationship might still be influenced by other factors. Here, we investigated taxonomic effects, the impact of genome completeness, genome size, and GC content. We found that read counts were most strongly influenced by taxonomy, followed by genome size, genome completeness, biomass, and repeat content (Figure 1c). We found no statistically significant effects of GC content on read assignment, although this was expected based on previous results with bacterial metagenomes (Browne et al., 2020).

There were consistently more reads assigned to some taxonomic groups than to others (Figure 1b, Table 2). The impact of taxonomy on sequencing reads recovery seems to be systemic, with some species having many reads in all mocks, some species having only few reads (Figure 1a), and one species was even missed due to the stringent filtering (Figure 2a). Species represented with low biomass in mocks were already found to result in false negatives in metagenomics (Bista et al., 2018), and *A*. *striculus* was indeed represented with a relatively low biomass in the mocks. However, low biomass alone does not explain the strong taxon effect on read assignment. We suspect that the most important cause for the strong taxon effects is likely caused by differences in DNA yields among different taxa (Sato et al., 2019; Schiebelhut et al., 2017; Tourlousse et al., 2021). Some taxa, e.g., oribatid mites, are very hardy, and their cuticles might present obstacles to tissue homogenization during DNA extractions. Indeed, the single false‐negative species was an oribatid mite. Cells of different taxa might react differently to extraction (Costea et al., 2017; Morgan et al., 2010), with some species consistently yielding lower‐quality DNA in lower quantities (or no DNA at all) than others (Schiebelhut et al., 2017). However, differential DNA extraction efficiency does not explain why soft‐bodied enchytraeids yield considerably less DNA than all other taxa (Figure 1b). Differences in DNA content relative to body size (or biomass) might be responsible for this: some taxa may contain higher amounts of DNA per unit biomass than others. The association of DNA content with body size can be positive or negative depending on the organism group (Gregory, 2001).

Strong taxonomic effects on biomass–read relationships are interesting not only for metagenomic but also for metabarcoding studies. It is generally assumed that primer mismatch is the most important source of taxonomically biased biomass–read relationships in metabarcoding (Collins et al., 2019; Lamb et al., 2019; Piñol et al., 2019). Our results suggest that taxon‐specific differences in DNA extraction efficiency and/or DNA content might also play a role in taxonomic bias. However, recognizing this bias is difficult in metabarcoding: both primer bias, and factors influencing extraction DNA yields are likely phylogenetically conserved. Parallel metabarcoding and metagenomic studies on the same mock communities are necessary to evaluate the relative importance of primer bias versus DNA yield in biomass–read relationships (e.g., Bista et al., 2018).

Despite considerable taxonomic effects, biomass was a statistically significant predictor of reads (Figure 1b, Table 2). This is in line with other metagenomic mock community studies on multicellular eukaryotes, such as benthic invertebrates (Bista et al., 2018) and pollen samples (Peel et al., 2019). The biomass effect on reads, although considerably smaller than taxon effects (Figure 1c), was still sufficient to reflect compositional differences among the four mock types (Figure 3). This confirms the suitability of shotgun metagenomics for a semi‐quantitative comparison of soil invertebrate communities.

We found that reference genome properties influence taxonomic assignments and read–biomass relationships, and that these need to be considered in metagenomic studies on eukaryotes. We showed that reference genomes size influences metagenomic assignments, with larger genomes attracting more reads than smaller genomes (Figure 1b). This is known from microbial studies where it was shown that average genome size of a microbial community influences metagenomic results (Beszteri et al., 2010). Repeat content is considered to positively influence genome size of eukaryotes, at least in the range of genome sizes of species analyzed here (Novák et al., 2020). We found a weak negative, but statistically significant effect of repeat content on metagenomic assignments (Figure 1b,c, Table 2). Repeat content and genome sizes were collinear. This collinearity also suggests that repetitive element abundance and repeat family composition may act as "hidden variables" in metagenomic read assignment. The effects of repetitive elements should be evaluated with highly contiguous and complete genomes which allow for an unbiased identification of the repetitive / non‐repetitive genome fractions. We found that genome completeness recorded as BUSCO scores may also influence metagenomic assignments, with more complete genomes attracting more reads. This suggests that reference genome assembly properties should also be considered in metagenomic assignments, even though previous findings show that even low‐coverage reference genomes can perform well (Sarmashghi et al., 2019). GC content of genomes might also influence metagenomic assignments (Browne et al., 2020), although in our case this effect was limited.

Considerable difference in sequencing effort (simulated by downsampling sequencing results to over 100th of original reads) had only minor influences on taxonomic identification, and on results about factors which influence read abundances and community composition (Figures S1, S2). We obtained highly similar results with as little as 100,000 reads per sample compared to the full‐sequencing effort (over 13 million reads per sample). This suggests that metagenomics of eukaryotic samples can be performed with low‐sequencing efforts and costs when the aim is the taxonomic profiling of samples. This is similar to results from bacterial communities (Gweon et al., 2019). Cost efficiency of metagenomics might even approach the costs of metabarcoding, although this probably depends on the complexity of communities. The low‐sequencing effort needed also suggests that relatively low‐yield long‐read eukaryote metagenomics, i.e., on Oxford Nanopore portable MinION sequencers, can be employed for eukaryotic metagenomics in areas which currently lack expensive short‐read sequencing infrastructure.

Mock community experiments of metagenomics are important to understand factors which influence species assignments and sequencing read abundances. However, experimental conditions are considerably simpler than conditions encountered in real community samples. Real samples likely contain more soil invertebrate species, including cryptic ones. Many of these species might not be present in genome databases, and this means that a large fraction of metagenomic reads might not be identifiable. We expect that this will rapidly change in the next years as biodiversity genomics initiatives cover more of eukaryotic diversity with reference quality genomes (Formenti et al., 2022; Lewin et al., 2022), and as phylogeny‐based assignment approaches are rapidly developing (Asnicar et al., 2020). Real samples will also contain higher numbers of bacteria and fungi. This is specifically true for eDNA samples where most reads likely originate from bacteria and fungi (Fierer et al., 2012). This means that sequencing depths will need to be higher when dealing with real communities, especially for eDNA samples. Other factors also need to be evaluated, such as release of eDNA be different taxa, legacy effects due to long‐term preservation of DNA in soils (Pedersen et al., 2015), or enzymatic inhibition and strategies for sample comparison (Hedman & Rådström, 2013). We are convinced that mock community experiments will remain important tools to understand these sources of variation.

### 4.1 Roadmap for future metagenomics on metazoans

Our results outline a roadmap for future shotgun metagenomic work on metazoan mock communities. In the wet lab, DNA extraction needs to be optimized and likely adapted to taxa of interest. An important component of this is to ensure the best possible homogenization of bulk samples, probably under cryogenic conditions. This is particularly important in order to detect species which yield low amounts of DNA, since this may frequently happen in more species‐rich natural communities. Differences in DNA content per unit biomass among and within major taxon groups should be evaluated and corrected for. In bioinformatics, assignment algorithms should be evaluated, adapted, and developed with eukaryotes in mind. The performance of distinct genomic regions (i.e., conventional marker genes, mitogenomes, coding regions, ultraconserved regions, and repeat elements) should be evaluated, especially with respect to false‐positive detections. Genome databases will likely remain incomplete for some time. An important direction is to evaluate how incomplete databases (i.e., databases not containing the target species, but congenerics or even less related species) perform in taxonomic assignments. It is also important to consider the completeness of the references genome assemblies, as more complete genomes will allow to assign more metagenomic reads. Genome completeness and genome size should be explicitly accounted for in analyses, for example, as predictors in generalized linear models.

## 5 CONCLUSION

Metagenomics is a promising alternative to metabarcoding also for eukaryotic communities, even at very low‐sequencing efforts. Although theory suggests that metagenomic reads should well‐represent biomass relationships in communities, differences among organisms related to DNA extraction efficiency and genome properties have strong influences on the biomass–read relationships. These effects need to be further investigated and quantified in parallel metabarcoding–metagenomic experiments. The effects of taxonomy, genome, and assembly properties should be considered in analyses. Generalized linear models provide an excellent opportunity for this. With affordable sequencing and increasingly accessible eukaryotic reference genomes, metagenomics is becoming a viable alternative to metabarcoding for describing community composition and structure.

## AUTHOR CONTRIBUTIONS

**Alexandra Schmidt:** Data curation (lead); Formal analysis (supporting); Investigation (lead); Methodology (lead); Project administration (lead); Software (lead); Visualization (supporting); Writing – original draft (lead); Writing – review & editing (supporting). **Clément Schneider:** Conceptualization (supporting); Methodology (supporting); Resources (supporting). **Peter Decker:** Conceptualization (supporting); Methodology (supporting); Resources (supporting); Writing – review & editing (supporting). **Karin Hohberg:** Methodology (supporting), Conceptualization (supporting); Resources (supporting); Writing – review & editing (supporting). **Jörg Römbke:** Resources (supporting). **Ricarda Lehmitz:** Conceptualization (supporting); Methodology (supporting); Resources (supporting); Writing – review & editing (supporting). **Miklos Balint:** Conceptualization (lead); Formal analysis (lead); Funding acquisition (lead); Investigation (supporting); Methodology (supporting); Resources (lead); Software (supporting); Supervision (lead); Validation (supporting); Visualization (lead); Writing – original draft (lead); Writing – review & editing (supporting).

## Supporting information

Figure S1‐S3‐Table S1

## Data Availability

Sequence data are available in GenBank (PRJNA758215). R scripts and inputs are available in FigShare (doi: https://doi.org/10.6084/m9.figshare.19711684.v1, https://doi.org/10.6084/m9.figshare.19657647.v2). Manuscript can be found at Dryad (https://doi.org/10.5061/dryad.15dv41p0k).
