# Correction to “Shotgun metagenomics of soil invertebrate communities reflects taxonomy, biomass, and reference genome properties”

**DOI:** 10.1002/ece3.10779

**Published:** 2023-12-20

Schmidt, A., Schneider, C., Decker, P., Hohberg, K., Römbke, J., Lehmitz, R., & Bálint, M. (2022). Shotgun metagenomics of soil invertebrate communities reflects taxonomy, biomass, and reference genome properties. *Ecology and Evolution*, *12*(6). https://doi.org/10.1002/ece3.8991

The ENA accession number in the Data Availability Statement is incorrect. It should read as:

Sequence data are available in GenBank (PRJEB45431). R scripts and inputs are available in Figshare (doi: https://doi.org/10.6084/m9.figshare.19711684.v1, https://doi.org/10.6084/m9.figshare.19657647.v2). Manuscript can be found at Dryad (https://doi.org/10.5061/dryad.15dv41p0k).

We apologize for this error.

