# Supplementary material for: Shotgun metagenomics of soil invertebrate communities reflects taxonomy, biomass, and reference genome properties
Source: Ecol Evol. 2022 Jun 6;12(6):e8991. doi: 10.1002/ece3.8991 (PMC9170594; doi:10.1002/ece3.8991)
Supplement: Supplementary file 1 — Figure S1‐S3‐Table S1 [file ECE3-12-e8991-s001.docx]

**Appendix**

# **Figures**


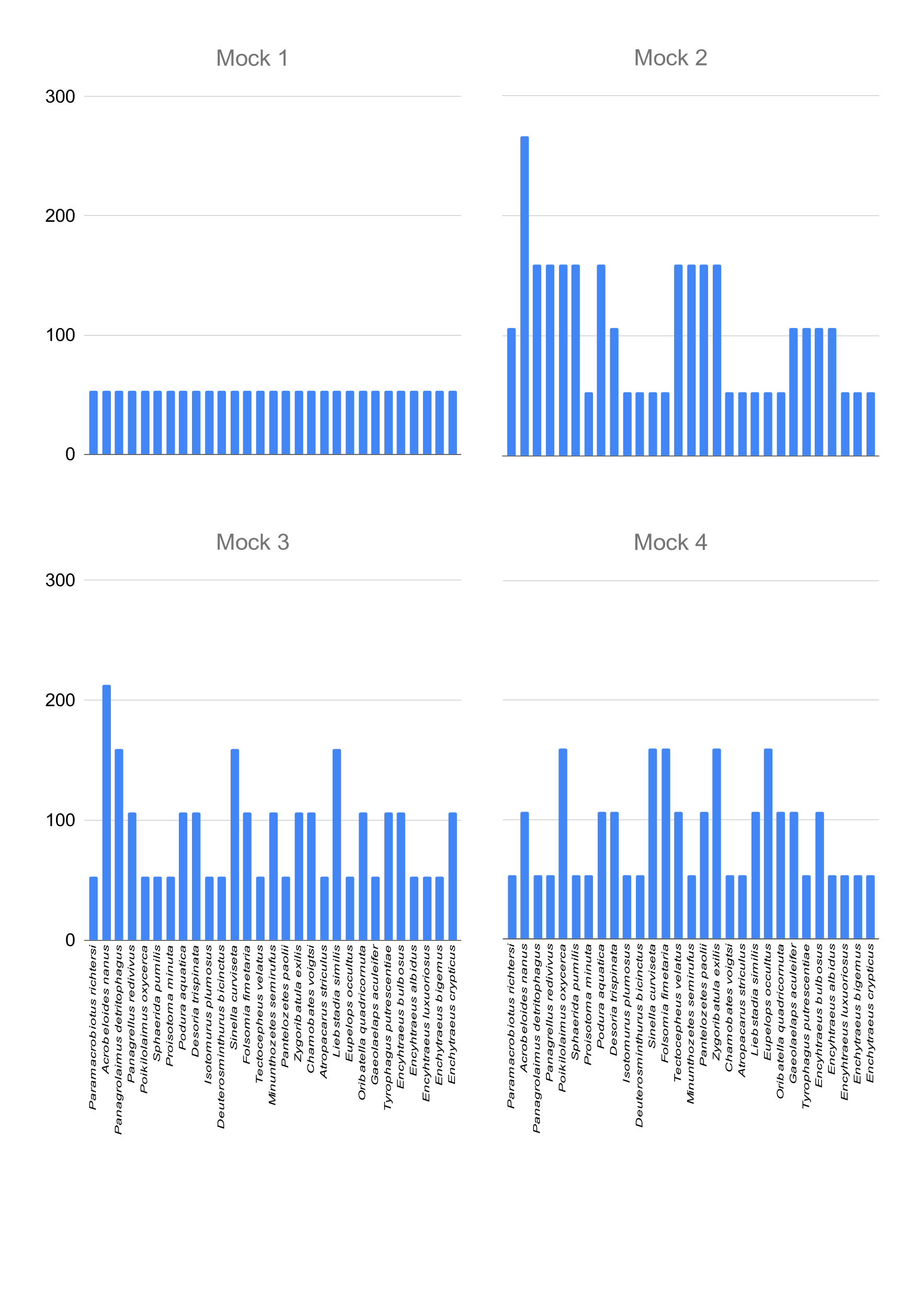


**Fig 1.** The mock community setups (Mock1-Mock4). Biomass ratios of species in four mock communities.


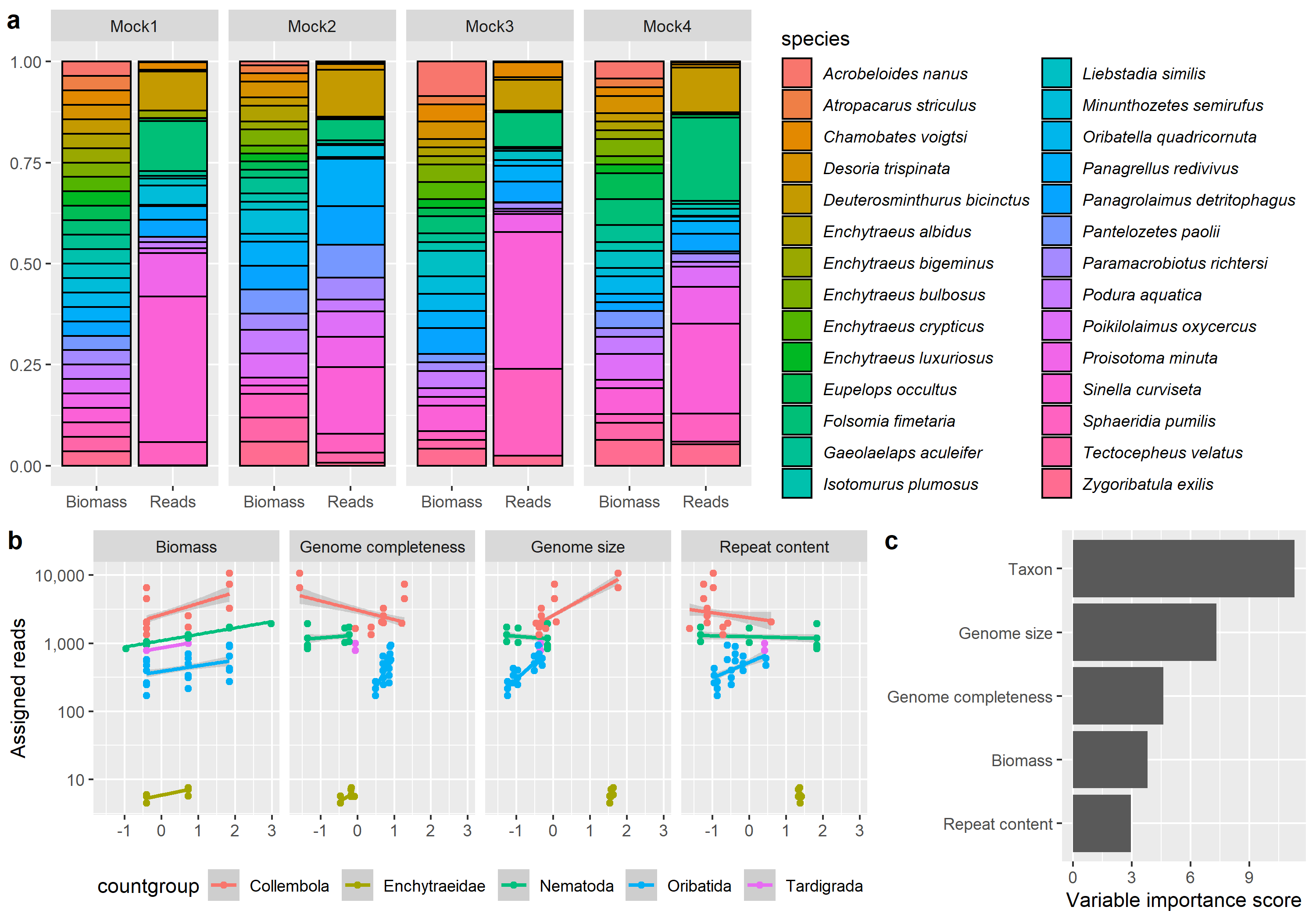


**Fig. 2**. The original dataset subsampled to 100K. a) Biomass ratios of taxa and sequencing reads assigned to these taxa in four mock communities. b) GLM-predicted effects of biomass, genome completeness, genome size and repeat content on taxonomically assigned metagenomic reads. c) Relative importance variables.


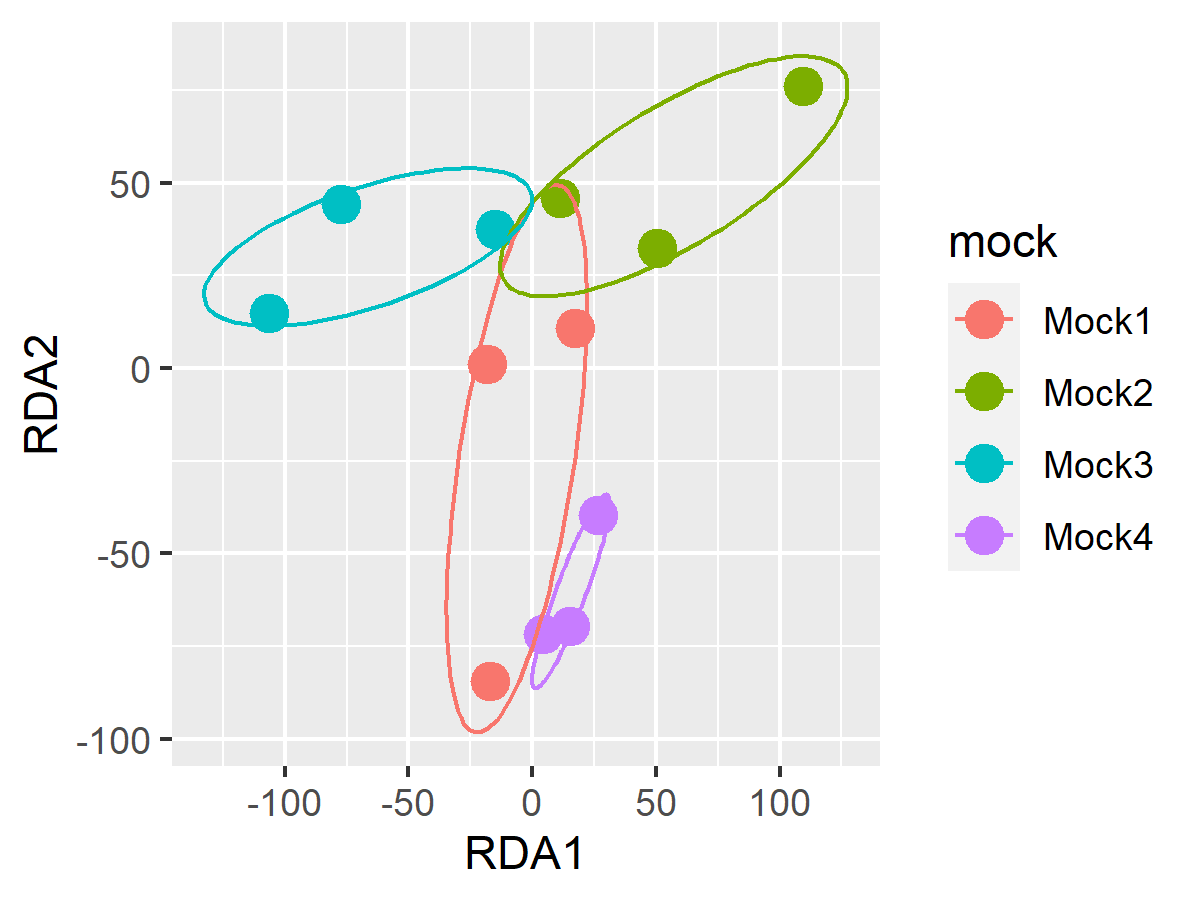


**Fig. 3.** The original dataset subsampled to 100K. Redundancy analysis ordination of mock community replicates along the taxonomically assigned metagenomic reads.

# Tables

**Table 1.** The list of soil invertebrate species and corresponding taxa (count group) included in the genome reference database.

| **Count group** | **Species** |
| --- | --- |
| Isopoda | *Philoscia muscorum* |
| Diplopoda | *Glomeris marginata* |
| Chilopoda | *Lithobius calcaratus* |
| Diplopoda | *Xestoiulus laeticollis* |
| Diplopoda | *Ommatoiulus sabulosus* |
| Diplopoda | *Cylindroiulus punctatus* |
| Diplopoda | *Polydesmus complanatus* |
| Chilopoda | *Cryptops parisi* |
| Diplopoda | *Julus scandinavius* |
| Diplopoda | *Polydesmus angustus* |
| Diplopoda | *Proteroiulus fuscus* |
| Chilopoda | *Pachymerium ferrugineum* |
| Chilopoda | *Strigamia transsilvanica* |
| Collembola | *Isotomurus pseudopalustris* |
| Collembola | *Isotoma caerulea* |
| Collembola | *Lepidocyrtus paradoxus* |
| Collembola | *Orchesella flavescens* |
| Collembola | *Orchesella bifasciata* |
| Isopoda | *Trachelipus rathkii* |
| Collembola | *Dicyrtoma fusca* |
| Collembola | *Dicyrtomina minuta* |
| Collembola | *Entomobrya muscorum* |
| Collembola | *Ceratophysella granulata* |
| Collembola | *Anurida granaria* |
| Collembola | *Isotomurus graminis* |
| Collembola | *Folsomia inoculata* |
| Collembola | *Sminthurinus aureus* |
| Chilopoda | *Haplophilus subterraneus* |
| Collembola | *Gisinianus flammeolus* |
| Collembola | *Pseudosinella alba* |
| Collembola | *Bilobella braunerae* |
| Collembola | *Vertagopus arboreus* |
| Collembola | *Lipothrix lubbocki* |
| Collembola | *Entomobrya nicoleti* |
| Collembola | *Pogonognathellus flavescens* |
| Chilopoda | *Henia vesuviana* |
| Collembola | *Dicyrtomina saundersi* |
| Collembola | *Lepidocyrtus cyaneus* |
| Collembola | *Willowsia buski* |
| Collembola | *Willowsia nigromaculata* |
| Diplura | *Campodea plusiochaeta* |
| Collembola | *Paratullbergia callipygos* |
| Isopoda | *Oniscus asellus* |
| Collembola | *Protaphorura quadriocellata* |
| Collembola | *Protaphorura armata* |
| Collembola | *Entomobrya cf arborea* |
| Collembola | *Agrenia bidenticulata* |
| Collembola | *Isotomurus maculatus* |
| Isopoda | *Porcellio scaber* |
| Collembola | *Hypogastrura burkilli* |
| Collembola | *Isotoma decorata* |
| Chilopoda | *Lithobius forficatus* |
| Chilopoda | *Lithobius microps* |
| Chilopoda | *Lithobius nodulipes* |
| Chilopoda | *Lithobius agilis* |
| Isopoda | *Trichoniscus pusillus* |
| Chilopoda | *Lithobius crassipesoides* |
| Chilopoda | *Lithobius crassipes* |
| Chilopoda | *Stenotaenia linearis* |
| Chilopoda | *Geophilus carpophagus* |
| Diplopoda | *Kryphioiulus occultus* |
| Diplopoda | *Choneiulus palmatus* |
| Isopoda | *Porcellio spinicornis* |
| Diplopoda | *Rossiulus vilnensis* |
| Diplopoda | *Ophyiulus pilosus* |
| Diplopoda | *Julus scanicus* |
| Diplopoda | *Melogona broelemanni* |
| Diplopoda | *Glomeris hexasticha* |
| Oribatida | *Adoristes ovatus* |
| Oribatida | *Edwardzetes edwardsi* |
| Collembola | *Sminthurinus signatus* |
| Collembola | *Spatulosminthurus flaviceps* |
| Collembola | *Bourletiella hortensis* |
| Collembola | *Folsomia candida* |
| Collembola | *Sminthurides aquaticus* |
| Collembola | *Isotoma anglicana* |
| Collembola | *Orthonychiurus folsomi* |
| Collembola | *Arrhopalites spinosus* |
| Collembola | *Entomobrya Typ multifasciata* |
| Oribatida | *Eueremaeus valkanovi* |
| Oribatida | *Eupelops torulosus* |
| Oribatida | *Hermanniella punctulata var. septentrionalis* |
| Oribatida | *Liacarus coracinus* |
| Oribatida | *Liacarus xylariae* |
| Oribatida | *Microtritia minima* |
| Oribatida | *Microzetes septentrionalis* |
| Oribatida | *Atropacarus striculus* |
| Oribatida | *Nanhermannia elegantula* |
| Oribatida | *Porobelba spinosa* |
| Oribatida | *Scheloribates initialis* |
| Gamasina | *Veigaia nemorensis* |
| Gamasina | *Parasitus lunulatus* |
| Gamasina | *Epicriopsis horridus* |
| Gamasina | *Pergamasus crassipes* |
| Oribatida | *Belba bartosi* |
| Nematoda | *Poikilolaimus oxycercus* |
| Collembola | *Folsomia fimetaria* |
| Collembola | *Deuterosminthurus bicinctus f. flava* |
| Oribatida | *Chamobates voigtsi* |
| Tardigrada | *Paramacrobiotus richtersi* |
| Collembola | *Sphaeridia pumilis* |
| Nematoda | *Panagrolaimus detritophagus* |
| Gamasina | *Pergamasus crassipes* |
| Gamasina | *Geholaspis longispinosus* |
| Gamasina | *Epicrius mollis* |
| Gamasina | *Phytoseiulus persimilis* |
| Gamasina | *Stratiolaelaps miles* |
| Gamasina | *Zercon vagabundus* |
| Chilopoda | *Strigamia crassipes* |
| Oribatida | *Camisia spinifer* |
| Oribatida | *Tectocepheus velatus* |
| Nematoda | *Heterodera schachtii* |
| Nematoda | *Meloidogyne incognita* |
| Nematoda | *Acrobeloides thornei* |
| Nematoda | *Acrobeloides obliquus* |
| Nematoda | *Acrobeloides cf. buchneri* |
| Nematoda | *Acrobeloides nanus* |
| Enchytraeidae | *Enchytraeus albidus* |
| Tardigrada | *Isohypsibius dastychi* |
| Collembola | *Megalothorax sp. 2* |
| Enchytraeidae | *Enchytraeus crypticus* |
| Enchytraeidae | *Enchytraeus bulbosus* |
| Enchytraeidae | *Enchytraeus luxuriosus* |
| Oribatida | *Punctoribates punctum* |
| Chilopoda | *Strigamia acuminata* |
| Lumbricina | *Dendrobaena attemsi* |
| Isopoda | *Ligidium hypnorum* |
| Diplopoda | *Nemasoma varicorne* |
| Collembola | *Ceratophysella sigillata* |
| Oribatida | *Carabodes subarcticus* |
| Collembola | *Ceratophysella scotica* |
| Collembola | *Orchesella cincta* |
| Collembola | *Pogonognathellus longicornis* |
| Collembola | *Tomocerus mixtus* |
| Collembola | *Supraphorura furcifera* |
| Collembola | *Isotomiella paraminor* |
| Collembola | *Hymenaphorura dentifera* |
| Collembola | *Micranurida granulata* |
| Oribatida | *Chamobates pusillus* |
| Collembola | *Friesea truncata* |
| Collembola | *Protaphorura glebata* |
| Collembola | *Xenyllodes armatus* |
| Pauropoda | *Allopauropus danicus* |
| Collembola | *Entomobrya corticalis* |
| Symphyla | *Scolopendrellopsis subnuda* |
| Collembola | *Arrhopalites* |
| Collembola | *Mesaphorura macrochaeta* |
| Symphyla | *Symphylella vulgaris* |
| Oribatida | *Ctenobelba pectinigera* |
| Diplura | *Campodea silvestrii* |
| Collembola | *Entomobrya nivalis* |
| Collembola | *Desoria olivacea* |
| Nematoda | *Discolaimus major* |
| Nematoda | *Rotylenchus robustus* |
| Nematoda | *Prionchulus punctatus* |
| Nematoda | *Anatonchus tridentatus* |
| Nematoda | *Aporcelaimellus obtusicaudatus* |
| Nematoda | *Rotylenchus robustus* |
| Nematoda | *Opisthodorylaimus sylphoides* |
| Oribatida | *Cultroribula bicultrata* |
| Nematoda | *Truxonchus dolichurus* |
| Nematoda | *Plectus parietinus* |
| Nematoda | *Mesodorylaimus bastiani* |
| Nematoda | *Anatonchus cf. genovi* |
| Oribatida | *Phthiracarus crinitus* |
| Oribatida | *Hafenrefferia gilvipes* |
| Isopoda | *Haplophthalmus danicus* |
| Diplopoda | *Megaphyllum sjaelandicum* |
| Diplopoda | *Tachypodoiulus niger* |
| Diplopoda | *Chordeuma sylvestre* |
| Diplopoda | *Mycogona germanica* |
| Chilopoda | *Geophilus truncorum* |
| Collembola | *Isotomurus palustris* |
| Collembola | *Hydroisotoma schaefferi* |
| Collembola | *Xenylla mediterranea* |
| Nematoda | *Plectus cf. velox* |
| Nematoda | *Eudorylaimus altherri* |
| Nematoda | *Pungentus monohystera* |
| Oribatida | *Eniochthonius minutissimus* |
| Nematoda | *Prismatolaimus dolichurus* |
| Oribatida | *Galumna obvia* |
| Oribatida | *Nanhermannia coronata cf.* |
| Oribatida | *Steganacarus magnus* |
| Oribatida | *Scheloribates latipes* |
| Nematoda | *Panagrellus redivivus* |
| Nematoda | *Discolaimus major* |
| Oribatida | *Heminothrus targionii* |
| Oribatida | *Nanhermannia nana* |
| Oribatida | *Pergalumna nervosa* |
| Oribatida | *Tectocepheus minor* |
| Collembola | *Desoria trispinata* |
| Collembola | *Lepidocyrtus lignorum* |
| Collembola | *Neelides folsomi* |
| Collembola | *Isotoma* |
| Collembola | *Neelus murinus* |
| Oribatida | *Allosuctobelba grandis* |
| Oribatida | *Carabodes femoralis* |
| Oribatida | *Hermannia gibba* |
| Oribatida | *Licneremaeus licnophorus* |
| Oribatida | *Mesoplophora pulchra* |
| Oribatida | *Metabelba pulverosa* |
| Oribatida | *Nothrus silvestris* |
| Collembola | *Sminthurinus sp.* |
| Enchytraeidae | *Cognettia cognettii* |
| Enchytraeidae | *Oconnorella tubifera* |
| Enchytraeidae | *Fridericia nemoralis* |
| Enchytraeidae | *Stercutus niveus* |
| Enchytraeidae | *Buchholzia appendiculata* |
| Enchytraeidae | *Enchytraeus norvegicus* |
| Collembola | *Allacma fusca* |
| Collembola | *Folsomia penicula* |
| Collembola | *Deuterosminthurus bicinctus* |
| Collembola | *Isotomurus unifasciatus* |
| Nematoda | *Plectus exinocaudatus* |
| Oribatida | *Phthiracarus laevigatus* |
| Oribatida | *Acrotritia duplicata* |
| Oribatida | *Minunthozetes semirufus* |
| Oribatida | *Nothrus palustris* |
| Oribatida | *Dissorhina ornata* |
| Oribatida | *Oribatella quadricornuta* |
| Oribatida | *Eupelops occultus* |
| Oribatida | *Zygoribatula exilis* |
| Oribatida | *Liebstadia similis* |
| Oribatida | *Tectocepheus sarekensis* |
| Oribatida | *Pantelozetes paolii* |
| Oribatida | *Achipteria nitens* |
| Diplopoda | *Megaphyllum projectum* |
| Diplopoda | *Polyxenus lagurus* |
| Diplopoda | *Polyzonium germanicum* |
| Collembola | *Ceratophysella denticulata* |
| Collembola | *Isotomiella minor* |
| Collembola | *Isotomurus plumosus* |
| Collembola | *Parisotoma notabilis* |
| Oribatida | *Hermanniella dolosa* |
| Oribatida | *Acrogalumna longipluma* |
| Oribatida | *Ceratozetes gracilis* |
| Oribatida | *Fuscozetes fuscipes* |
| Oribatida | *Galumna lanceata* |
| Oribatida | *Gustavia microcephala* |
| Diplopoda | *Cylindroiulus caeruleocinctus* |
| Diplopoda | *Leptoiulus proximus* |
| Diplopoda | *Strongylosoma stigmatosum* |
| Diplopoda | *Unciger foetidus* |
| Collembola | *Heteromurus nitidus* |
| Aves | *Gallus gallus* |
| Collembola | *Proisotoma minuta* |
| Collembola | *Protaphorura fimata* |
| Collembola | *Protaphorura tricampata* |
| Collembola | *Lepidocyrtus violaceus* |
| Collembola | *Sinella curviseta* |
| Collembola | *Tetrodontophora bielanensis* |
| Lumbricina | *Aporrectodea caliginosa* |
| Collembola | *Thalassaphorura encarpata* |
| Collembola | *Tomocerus minor* |
| Collembola | *Pseudisotoma sensibilis* |
| Oribatida | *Achipteria coleoptrata* |
| Oribatida | *Platynothrus peltifer* |
| Nematoda | *Aphelenchus avenae* |
| Oribatida | *Nanhermannia comitalis* |
| Collembola | *Isotoma viridis* |
| Oribatida | *Hypochthonius rufulus* |
| Nematoda | *Phasmarhabditis papillosa* |
| Chilopoda | *Geophilus flavus* |
| Chilopoda | *Schendyla nemorensis* |
| Oribatida | *Conchogneta dalecarlica* |
| Enchytraeidae | *Enchytraeus bigeminus* |
| Oribatida | *Oppiella nova* |
| Chilopoda | *Lithobius mutabilis* |
| Collembola | *Sminthurinus elegans* |
| Collembola | *Disparrhopalites patrizii* |
| Collembola | *Sminthurinus bimaculatus* |
| Oribatida | *Euzetes globulus* |
| Oribatida | *Malaconothrus monodactylus* |
| Nematoda | *Dolichorhabditis dolichura* |
| Oribatida | *Quadroppia maritalis* |
| Collembola | *Podura aquatica* |
